# Global Challenges: Bridging Business, Law, Science, Design, and Engineering for Sustainability

**DOI:** 10.1002/gch2.202500273

**Published:** 2025-06-30

**Authors:** Rui Torres de Oliveira, Agnes Toth‐Peter, Leonie Barner

**Affiliations:** ^1^ IPA‐Deakin SME Research Centre, Faculty of Business and Law Deakin University Melbourne 3220 Australia; ^2^ ARC Centre of Excellence in Synthetic Biology The University of Queensland Brisbane 4067 Australia; ^3^ School of Chemistry and Physics Queensland University of Technology Brisbane 4100 Australia

The 21st century faces a planetary crisis, where biodiversity loss, climate change, resource depletion (caused by human activities), technological disruptions, and economic inequality intersect to challenge *sustainable development*.^[^ ^1^, ^2^ ^]^ These interconnected issues have brought *sustainability* to the forefront as a critical concept.^[^ ^3^ ^]^ What began as a focus on environmental issues has evolved into a rich, multidimensional concept, and *sustainability* now integrates economic and social considerations alongside ecological concerns, giving rise to a broad and dynamic range of sustainability discourses. Sustainability has become a central ambition across policy, business, and academia, with a substantial and enduring influence that continues to shape national and international agendas and drive action across sectors.^[^ ^4^ ^]^

Sustainability and sustainable development emphasise that human development and economic growth should occur without threatening people, animals, ecosystems, or the Earth's stability.^[^ ^5^ ^]^ However, current indicators starkly challenge this ideal. In 2024, Earth Overshoot Day, marking the date when resource consumption exceeds Earth's capacity to regenerate those resources, fell on August 1, highlighting the ongoing ecological deficit.^[^ ^6^ ^]^ Further, as of 2023, six of the nine planetary boundaries have been transgressed, placing humanity outside the Earth's safe operating space. These include climate change, biodiversity loss, freshwater use, land change, biogeochemical flows, and novel entities.^[^ ^2^ ^]^

Tackling these sustainability challenges is complex, and it requires collaboration across fields, as no single discipline can fully grasp or resolve these systemic issues and develop innovative, viable, and practical solutions. Despite the recognised need for interdisciplinary approaches to help solve these complex problems, the integration of different disciplines remains bound to barriers and challenges.^[^ ^7^, ^8^ ^]^

In the context of research and academia, the concept of sustainability appeared in the mid‐1980s.^[^ ^9^ ^]^ However, disciplines such as business, economics, law, science, design, and engineering have traditionally operated within distinct silos, with limited potential for cross‐disciplinary synergy. As a result, the interest and focus of these fields often differ, with each discipline prioritizing unique questions to shape *their* approach to sustainability. For instance, business scholars focus on sustainability through the lenses of management, economics, international business, strategy, organizational and consumer behavior, and economic viability^[^ ^10^, ^11^ ^]^ while science scholars explore environmental and ecological dimensions through empirical observation, modeling, and experimental analysis.^[^ ^12^, ^13^, ^14^ ^]^ Engineering researchers emphasize technological advancements, process optimization, and material efficiency to achieve sustainable engineering processes,^[^ ^15^, ^16^ ^]^ and legal scholars play a crucial role in sustainability by shaping regulatory frameworks, enforcing compliance, and developing policies that balance economic development with environmental and social responsibility.^[^ ^17^, ^18^ ^]^ Despite the common goal ahead and the complementary nature of these perspectives, differences in research methodologies, theoretical frameworks, and publication norms often hinder meaningful collaboration.^[^ ^19^ ^]^

A fundamental challenge in integrating business, economics, law, science, design, and engineering research stems from their distinct epistemological and methodological traditions, as “two opposing disciplinarians can look at the same thing and not see the same thing.”^[20, p. 11]^ Business research often adopts constructivist or interpretivist epistemologies, exploring sustainability through qualitative methodologies such as case studies, ethnographies, and discourse analysis, which serve theory elaboration and generation rather than testing.^[^ ^21^ ^]^ Similarly, studies in law often integrate empirical methods, including qualitative case studies and comparative legal analysis, to assess the effectiveness and implementation of sustainability laws in practice.^[^ ^22^ ^]^ In contrast, science largely relies on positivist and post‐positivist approaches, applying empirical methods, field experiments, and computational modeling to analyze environmental changes using general rules, principles, and systemic techniques.^[^ ^23^ ^]^ Engineering research is similarly rooted in positivist traditions, relying on quantitative methods, controlled experiments, and simulation modeling to develop technological solutions for sustainability.^[^ ^24^ ^]^

However, these divergent research paradigms make it difficult to establish common ground. While each field aims to contribute to theory‐building, they differ in how theories are developed, as each discipline relies on a distinct set of observational categories and meanings.^[^ ^20^ ^]^ For instance, business prioritizes strategic implications, science focuses on fundamental discoveries and environmental impacts, engineering emphasizes technical feasibility and optimization, and legal research examines regulatory frameworks, governance mechanisms, and compliance structures to ensure the enforceability and effectiveness of sustainability policies. Therefore, at the beginning of any interdisciplinary collaboration, it is recommended to establish a common ground regarding the language and terms to achieve a shared framing of the problems, and learn the key terms of each other's disciplines as well as research methodologies.^[^ ^8^ ^]^

The epistemological and methodological divergence also extends to publication norms and peer review processes,^[^ ^25^ ^]^ with each discipline requiring research outcomes to conform to its own established standards.^[^ ^26^ ^]^ Business and economics research follows a rigorous double‐blind review process, which was found to have lower acceptance rates and extensive and more critical revisions that often lead to publication cycles spanning up to three years. Science and engineering research, on the other hand, frequently adopt single‐blind (only hiding the reviewer's identity from the author) or open review processes, leading to much shorter publication timelines. Nonetheless, the single‐blind process can potentially create biases, such as male corresponding authors having a significantly higher acceptance rate.^[^ ^25^ ^]^ These differences in review systems and expectations can create barriers for interdisciplinary research, as scholars from one field may struggle to meet the methodological and editorial standards of the other. As a result, producing knowledge is often impacted by publishing norms of specific journals, instead of focusing on what is most critical for advancing knowledge and informing practice and policy.^[^ ^26^ ^]^

Furthermore, interdisciplinary collaboration remains challenging due to differences in funding structures, research incentives, and industry engagement models. Science and engineering research are often driven by government and industry‐funded projects aimed at solving specific technical or ecological problems, whereas business and economics research is more likely to be supported by academic grants that prioritize theoretical contributions. In addition, scientific and engineering research typically requires significantly greater funding compared to research in business or law,^[^ ^27^ ^]^ due to their reliance on resource‐intensive and tangible assets like infrastructure, laboratories, equipment, and research staff. Moreover, even when researchers embark on interdisciplinary projects, they often face difficulties in obtaining funding, mainly due to funding agencies’ distinct programmatic priorities.^[^ ^28^ ^]^ These misalignments have been leading to siloed projects and hindering interdisciplinary collaborations.

However, these misalignments also show an untapped opportunity to learn from one another and integrate technological innovation and scientific discoveries with business strategy, design, and policy. This highlights the need for collaboration between business, economics, law, science, design, and engineering researchers to develop comprehensive sustainability solutions that are technologically feasible, scientifically sound, and economically viable, through an integrated approach.

Moreover, bridging this disciplinary divide also requires stronger partnerships between academia, industry, and policymakers to ensure that industry‐relevant research integrates business, science, design, and engineering perspectives and attempts more practical sustainability solutions. For example, business and law scholars can contribute insights into market adoption, regulatory and legislative challenges, and consumer behavior. At the same time, science scholars provide critical data on environmental impact and natural resource management, and engineers and designers are able to develop sustainable production processes, renewable energy systems, and eco‐efficient designs. Case studies of successful collaborations and design thinking, in particular, can provide valuable models for future research and highlight best practices for integrating interdisciplinary expertise in sustainability.

*Global Challenges* aims to address exactly these differences by providing a platform for interdisciplinary dialogue and collaboration between business, economics, law, science, design, and engineering researchers, among others. By bringing together diverse epistemological and methodological approaches and perspectives, we aim to harmonise the disciplinary differences and integrate such insights to accelerate the advancement of sustainability knowledge and create actionable solutions to pressing environmental and societal issues. As one of the leading journals in *interdisciplinary* research, with this focus at the core of the journal, we encourage contributions that focus on the cross‐cutting nature of sustainability and propose frameworks for integrating, among others, business, economics, law, science, design, and engineering insights whenever we can. This does not mean that we need to integrate all of these different disciplines at once, but rather have the critical thinking to bring to the table the *right* specialists depending on the problem at hand. Thus, at *Global Challenges*, we are interested in theoretical, design thinking, real‐world examples, and commentary pieces that demonstrate successful interdisciplinary collaborations and introduce novel methodologies that bridge the gap between these fields for sustainable development.

In a nutshell, *Global Challenges* aspires to establish new research paradigms that move beyond traditional and siloed disciplinary boundaries and facilitate more effective, timely, and scalable sustainability solutions, while showing respect for the unique nature and differences of disciplines where authors come from. In this way, we welcome the ideas from diverse fields and offer opportunities to critically examine and integrate them to achieve a higher potential impact. Within the sustainability focus, *Global Challenges* invites high‐quality conceptual, empirical, and methodological papers that underline the complexity and interconnected nature of sustainability issues. Submissions are encouraged to provoke new insights, critically examine existing approaches, including governance and regulatory structures, and propose innovative frameworks, methodologies, new partnerships, and funding avenues.

## Conflict of Interest

The authors declare no conflict of interest.
